# The N-terminus of the *Aspergillus fumigatus* group III hybrid histidine kinase TcsC is essential for its physiological activity and targets the protein to the nucleus

**DOI:** 10.1128/mbio.01184-24

**Published:** 2024-06-04

**Authors:** Anna Vincek, Anja Wolf, Astrid Thomas, Frank Ebel, Sebastian Schruefer

**Affiliations:** 1 Institute for Infectious Diseases and Zoonoses, LMU Munich, Germany; Karlsruhe Institute of Technology (KIT), Karlsruhe, Germany

**Keywords:** *Aspergillus fumigatus*, TcsC, HOG pathway, group III hybrid histidine kinase, nuclear localization sequence, Ypd1, SakA

## Abstract

Group III hybrid histidine kinases are fungal-specific proteins and part of the multistep phosphorelay, representing the initial part of the high osmolarity glycerol (HOG) pathway. TcsC, the corresponding kinase in *Aspergillus fumigatus,* was expected to be a cytosolic protein but is targeted to the nucleus. Activation of TcsC by the antifungal fludioxonil has lethal consequences for the fungus. The agent triggers a fast and TcsC-dependent activation of SakA and later on a redistribution of TcsC to the cytoplasm. High osmolarity also activates TcsC, which then exits the nucleus or concentrates in spot-like, intra-nuclear structures. The sequence corresponding to the N-terminal 208 amino acids of TcsC lacks detectable domains. Its loss renders TcsC cytosolic and non-responsive to hyperosmotic stress, but it has no impact on the antifungal activity of fludioxonil. A point mutation in one of the three putative nuclear localization sequences, which are present in the N-terminus, prevents the nuclear localization of TcsC, but not its ability to respond to hyperosmotic stress. Hence, this striking intracellular localization is no prerequisite for the role of TcsC in the adaptive response to hyperosmotic stress, instead, TcsC proteins that are present in the nuclei seem to modulate the cell wall composition of hyphae, which takes place in the absence of stress. The results of the present study underline that the spatiotemporal dynamics of the individual components of the multistep phosphorelay is a crucial feature of this unique signaling hub.

**IMPORTANCE:**

Signaling pathways enable pathogens, such as *Aspergillus fumigatus*, to respond to a changing environment. The TcsC protein is the major sensor of the high osmolarity glycerol (HOG) pathway of *A. fumigatus* and it is also the target of certain antifungals. Insights in its function are therefore relevant for the pathogenicity and new therapeutic treatment options. TcsC was expected to be cytoplasmic, but we detected it in the nucleus and showed that it translocates to the cytoplasm upon activation. We have identified the motif that guides TcsC to the nucleus. An exchange of a single amino acid in this motif prevents a nuclear localization, but this nuclear targeting is no prerequisite for the TcsC-mediated stress response. Loss of the N-terminal 208 amino acids prevents the nuclear localization and renders TcsC unable to respond to hyperosmotic stress demonstrating that this part of the protein is of crucial importance.

## INTRODUCTION

Group III hybrid histidine kinases (HHK) are fungal-specific signaling molecules that control responses to different stress conditions. All HHK possess a highly conserved C-terminal signaling module and a diverse N-terminal sensing module. For group III HHK, this part consists of several HAMP domains (1). These domains were first identified in histidine kinases, adenylate cyclases, methyl-accepting proteins, and phosphatases of bacteria. Each HAMP domain is an alpha-helical region of approximately 50 amino acids (aa). Structural data, which are available for some bacterial poly-HAMP domains, indicate that conformational changes mediate the signal transmission process (2, 3). Their modular architecture makes poly-HAMP domains versatile tools for the processing of various environmental signals. Fungal HHK can be divided into 11 different groups depending on their sensing modules (4, 5). HHK of groups VI and III have been functionally linked to the high osmolarity glycerol (HOG) pathway and play an important role in the adaptation to hyperosmotic stress, but are furthermore involved in responses to a wide array of other stress conditions (6).

TcsC is the only group III HHK of *Aspergillus fumigatus*, a pathogenic mold that menaces severely immunocompromised patients. Deletion of the *tcs*C gene impairs the ability of the mutant to adapt to hyperosmotic and high salt stress. Even under ambient conditions, the Δ*tcs*C colonies are smaller and have a distinct morphology. A general feature of mutants that lack group III HHK is their resistance to the antifungal pyrrolnitrin and its chemical derivative fludioxonil (7). Further phenotypes of the Δ*tcs*C mutant include a sporulation defect under certain stress conditions and an increased resistance to cell wall stressors, such as Calcofluor white (CFW) and Congo red (CR) (8). Treatment of *A. fumigatus* with fludioxonil results in an increase of the internal glycerol concentration and a dramatic swelling of the hyphal cells. This characteristic ballooning is accompanied by the formation of additional septa, closure of septal pores, an elevated number of nuclei per compartment, and a profound reorganization of the cell wall (8, 9). The outcome of this process is a rupture of the cell envelope and a release of the cytoplasmic content. This ability to kill fungal cells and the fact that group III HHK do not exist in mammals makes them attractive targets for the development of novel antifungals (10).

Recent data showed that TcsC executes its activity via the histidine phosphotransfer (Hpt) protein Ypd1 and the downstream response regulators Skn7 and SskA (11, 12); all four proteins form a so-called multistep phosphorelay (13). Our current knowledge of the mechanisms that activate TcsC and other group III HHK is still in its infancy. The localization of these proteins within the fungal cells is a relevant feature in this context, but to date only two group III HHK have been characterized in this respect: DhNIK1 of *Debaryomyces hansenii* was detected in the cytoplasm, but not in the nucleus after heterologous expression in *Saccharomyces cerevisiae* (14), whereas Nik1 of *Candida guilliermondii* was distributed throughout in the whole nucleo-cytoplasm (15). In a previous study, we analyzed the intracellular localization of a set of N-terminally truncated TcsC proteins and detected all of them in the cytoplasm (16). This and the absence of predictable targeting motifs suggested that TcsC is a cytoplasmic protein. However, our data show that in the absence of stress, TcsC resides in the nucleus and this was the starting point for the current study.

## RESULTS

**Fig 1:**
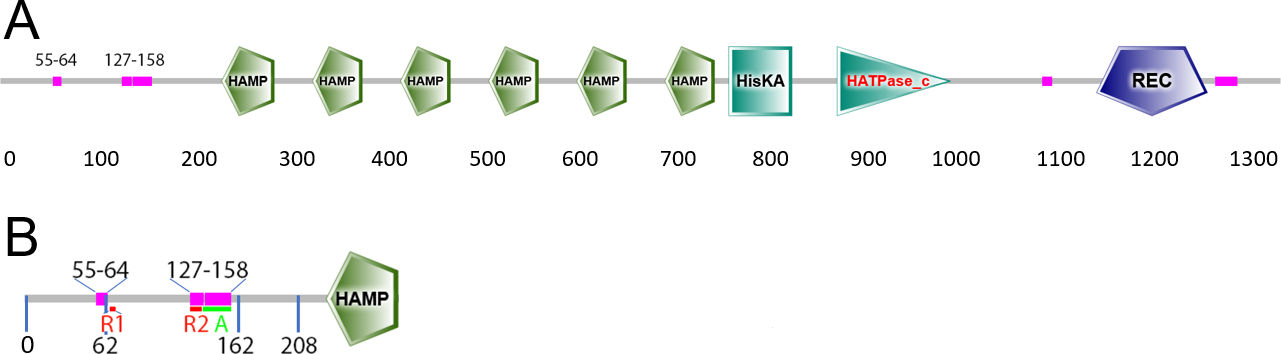
Schematic representation of the TcsC protein of *A. fumigatus*. The complete protein is depicted in panel A. The domains and regions are predicted using the SMART algorithm. The indicated sequences 55–64 and 127–158 are regions of low complexity. Panel B shows only the N-terminus. The basic regions 1 and 2 (R1 and R2) are indicated in red. The acidic region (**A**) is depicted in green. Residues that represent borders of truncated proteins are also indicated.

TcsC harbors several domains that are depicted in Fig. 1A. In this study, we have characterized the N-terminal part that consists of 208 aa; the different regions and key residues of this part of the TcsC protein are summarized in Fig. 1B.

The function of a protein is often influenced by its intracellular localization. TcsC is predicted by the WoLF PSORT algorithm to be a cytosolic protein (cyto: 14), but the second-best option is nucleo-cytoplasmic (cyto_nucl: 9.5). PSORT II, which includes the “NNCN: Reinhardt’s method for cytoplasmic/nuclear discrimination,” predicts a cytoplasmic localization for TcsC with a score of 76.7.

**Fig 2:**
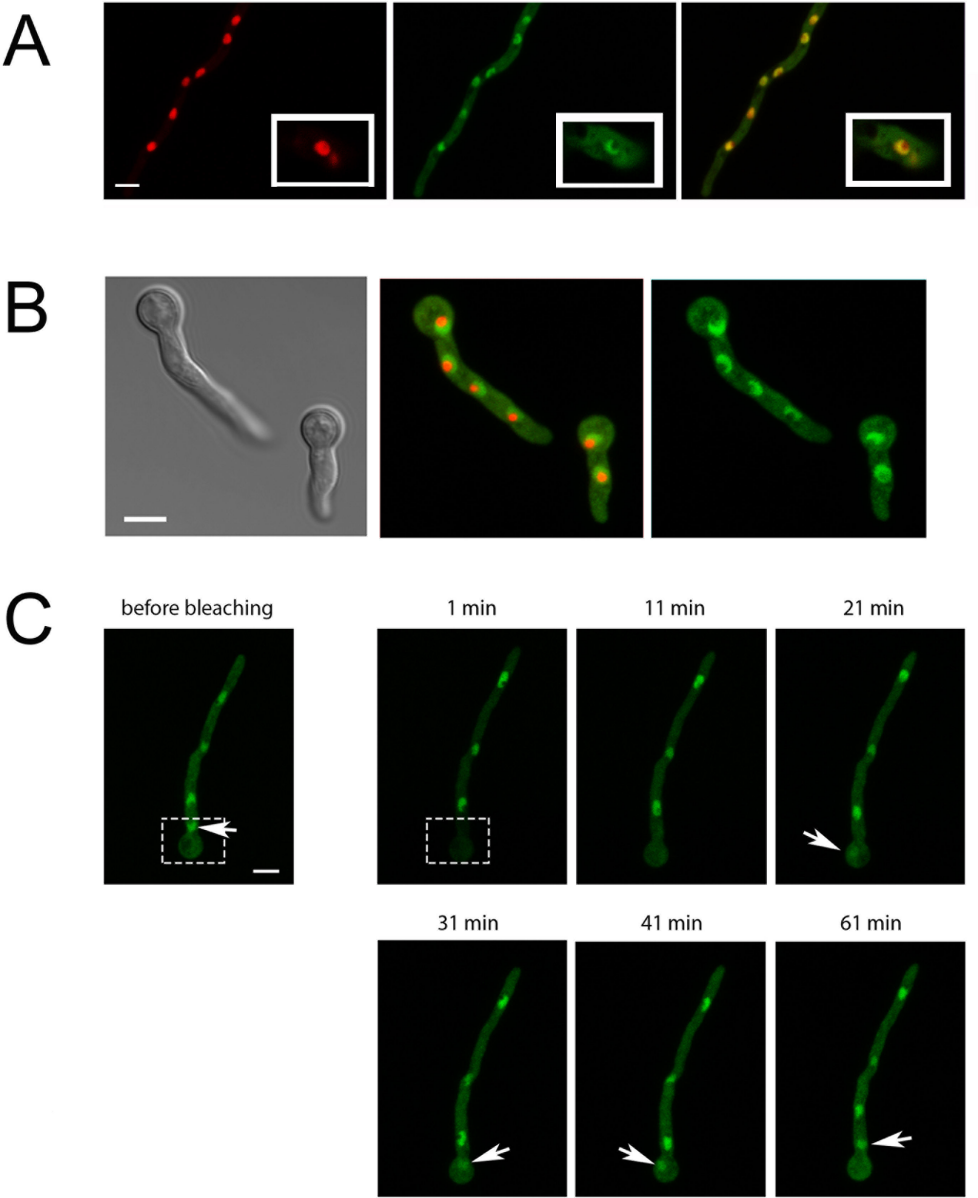
Localization of GFP-TcsC in *A. fumigatus* strain AfS35. Panel A shows the localization of the nuclear marker RFP-StuA in red, the distribution of GFP-TcsC in green, and an overlay of both channels. The insets show enlargements of one nucleus. Panel B shows germlings co-expressing GFP-TcsC and RFP-fibrillarin. The results of a bleaching experiment are shown in panel C. The initial fluorescence image (before bleaching) shows a hypha with four nuclei. The area that was bleached is boxed. The other images show the green fluorescence at the indicated time points post-bleaching. The position of the bleached nucleus is indicated by arrows. All fluorescence images represent maximum projections, only the bright field image in panel B depicts one optical plane. The bars in the leftmost panels of A, B, and C represent 5 µm each and are valid for all images of the respective panel.

Based on this information, we expected TcsC to be a cytosolic protein, but a GFP-TcsC fusion was enriched in distinct organelle-like structures that, by co-expression with RFP-StuA, were identified as nuclei (Fig. 2A). A small part of the nucleus contained only RFP-StuA, but no GFP-TcsC (Fig. 2A, enlargements). In their size and shape, these intra-nuclear structures resembled nucleoli, and co-expression of GFP-TcsC and fibrillarin (Afu1g14220)-RFP confirmed this (Fig. 2B). Hence, GFP-TcsC is recruited to the nucleus, but it is excluded from the nucleoli.

To analyze the dynamics of GFP-TcsC, we bleached a hyphal region containing one nucleus (Fig. 2C, boxed region). This treatment resulted in a loss of GFP-fluorescence in this nucleus and a strongly reduced fluorescence in the whole cytoplasm (Fig. 2C, 1 min). The bleached nucleus regained some fluorescence after 21 min, but it took another 40 min until the level was comparable to that of non-bleached nuclei (Fig. 2C). These data demonstrate a slow exchange of GFP-TcsC molecules between the cytoplasmic and the nuclear compartment.

**Fig 3:**
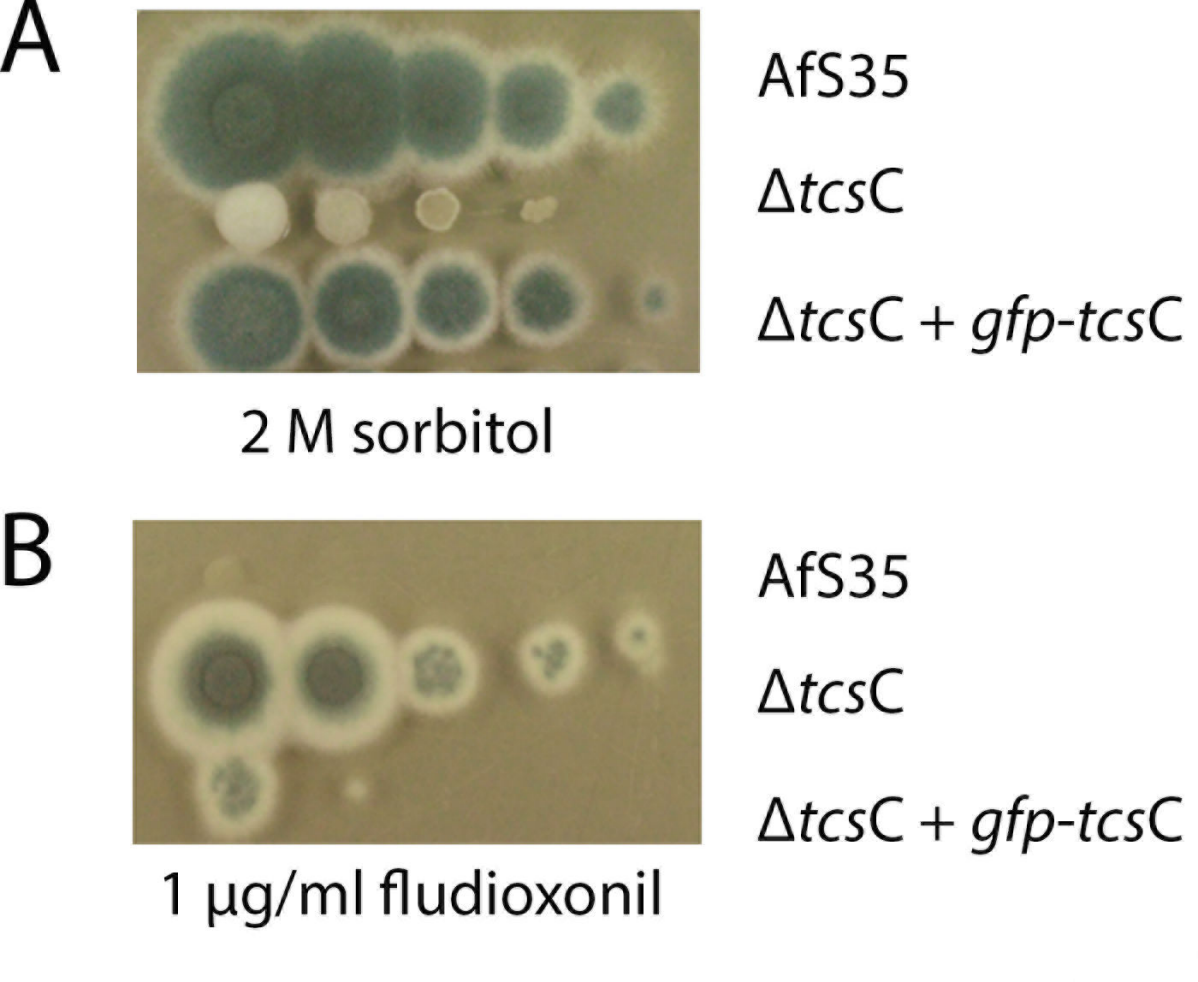
Analysis of the biological activity of GFP-TcsC. Drop dilution assays were performed to compare the ability of the parental strain AfS35, the Δ*tcs*C mutant, and the Δ*tcs*C mutant expressing GFP-TcsC to grow in the presence of 2 M sorbitol (panel A) and 1 µg/mL fludioxonil (panel B). The data demonstrate that the fusion protein can restore wild-type-like responses to hyperosmotic stress and the antifungal agent fludioxonil.

To analyze whether GFP-TcsC is functional, we expressed it in a Δ*tcs*C mutant and analyzed the phenotypes of the resulting strain. As expected from previous studies, the growth of the Δ*tcs*C mutant was strongly impaired in the presence of 2 M sorbitol (Fig. 3A), but expression of GFP-TcsC resulted in colonies that were comparable to that of the wild type. GFP-TcsC also restored the sensitivity of the complemented mutant to fludioxonil (Fig. 3B). Fludioxonil-treated hyphae of the complemented strain showed a characteristic ballooning, a shedding of galactomannan and a much stronger CFW staining (data not shown). Taken together, these results demonstrate that GFP-TcsC can functionally complement the Δ*tcs*C mutant.

A nuclear localization of GFP-TcsC was also observed in the Δ*tcs*C mutant (Fig. S1A) indicating that the presence of native TcsC is no prerequisite for the targeting of the fusion protein. Moreover, the position of the GFP moiety in the fusion had no influence on the localization, since TcsC-GFP showed a similar nuclear enrichment as GFP-TcsC (Fig. S1B).

**Fig 4:**
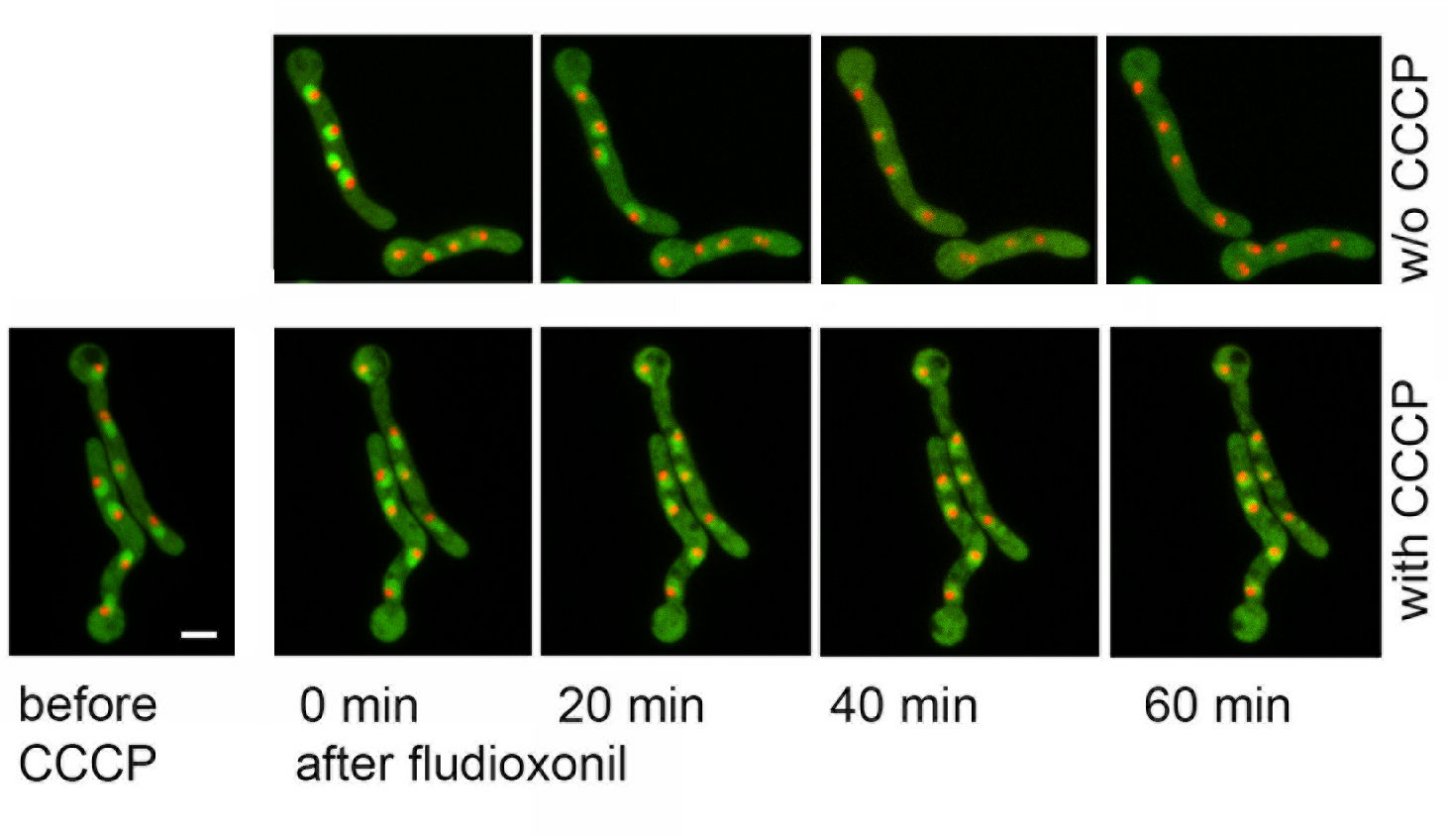
Fludioxonil triggers an energy-dependent translocation of GFP-TcsC to the cytoplasm. Short hyphae of *A. fumigatus* expressing GFP-TcsC and fibrillarin-RFP were incubated with 1 µg/mL fludioxonil (upper set of images) or they were pretreated with 200 µM carbonyl cyanide m-chlorophenyl hydrazine (CCCP) for 5 min and then exposed to fludioxonil (lower set of images). Images were taken at the indicated times after addition of fludioxonil and show maximum projections of stacks of confocal images. The bar indicates 5 µm and is valid for all images.

Using live cell microscopy, we analyzed the localization of GFP-TcsC in fludioxonil-treated hyphae over time. The nuclear enrichment of GFP-TcsC vanished after approximately 40 to 60 min (Fig. 4, upper set of images). A truncated TcsC protein comprising aa 1–746, which comprises the N-terminus and all HAMP domains, but lacks the signaling module, is also targeted to the nucleus (Fig. S2B). This fusion protein showed no redistribution in response to fludioxonil (Fig. S2D) demonstrating that the signaling module is essential for this process.

We next analyzed whether the nuclear targeting of GFP-TcsC is energy-dependent. Carbonyl cyanide m-chlorophenyl hydrazine (CCCP) inhibits oxidative phosphorylation and at a concentration of 200 µM, it causes an immediate growth arrest of *A. fumigatus* hyphae (data not shown). Fludioxonil treatment of hyphae expressing GFP-TcsC and RFP-fibrillarin resulted in the translocation of GFP-TcsC from the nucleus to the cytoplasm, whereas RFP-fibrillarin remained in the nucleoli (Fig. 4, upper set of images). Pretreatment of hyphae with CCCP prevented the fludioxonil-induced translocation of GFP-TcsC to the cytoplasm (Fig. 4, lower set of images) indicating that this process is energy-dependent.

**Fig 5:**
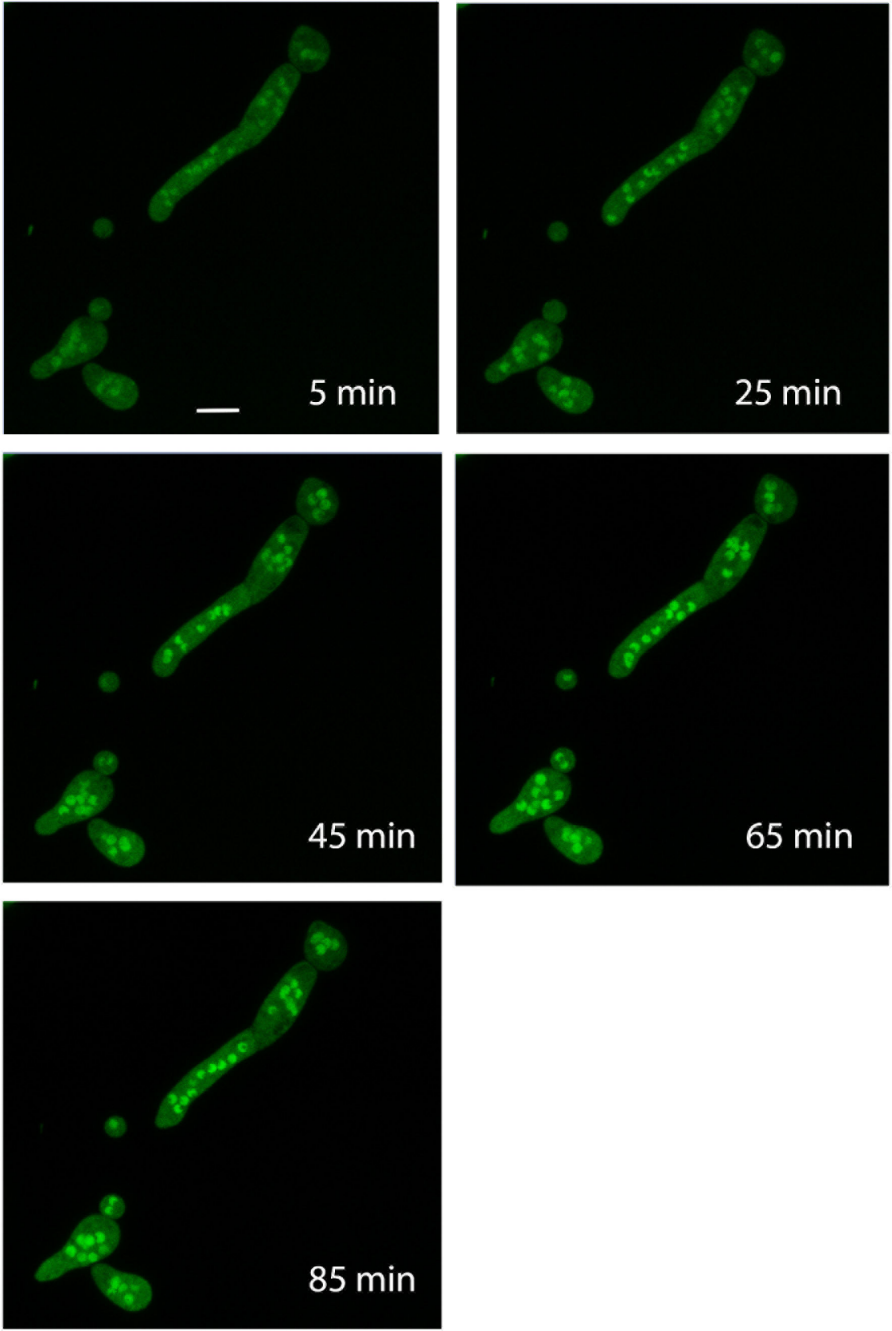
After the removal of fludioxonil, GFP-TcsC migrates back to the nuclei. Hyphae of *A. fumigatus* strain AfS35 expressing GFP-TcsC were pretreated with fludioxonil (1 µg/mL) for 3 h. The medium was then replaced by fresh Aspergillus minimal medium (AMM) without fludioxonil and the sample was analyzed by life cell microscopy at the indicated times after removal of fludioxonil. The images show maximum projections of stacks of confocal images. The bar in the first panel represents 5 µm and is valid for all other panels. The increased number of nuclei is typical for fludioxonil-treated hyphae.

To investigate whether the fludioxonil-induced redistribution of TcsC is reversible, hyphae were pretreated with fludioxonil for 3 h, washed, and then analyzed by live cell microscopy. The GFP-TcsC fluorescence in the nuclei was initially weak, but became more and more prominent and after approximately 45 min the nuclear enrichment reached a level that resembled that of untreated control hyphae (Fig. 5 and data not shown). This indicates that the fludioxonil-induced migration of GFP-TcsC is reversible and linked to the activation of TcsC.

**Fig 6:**
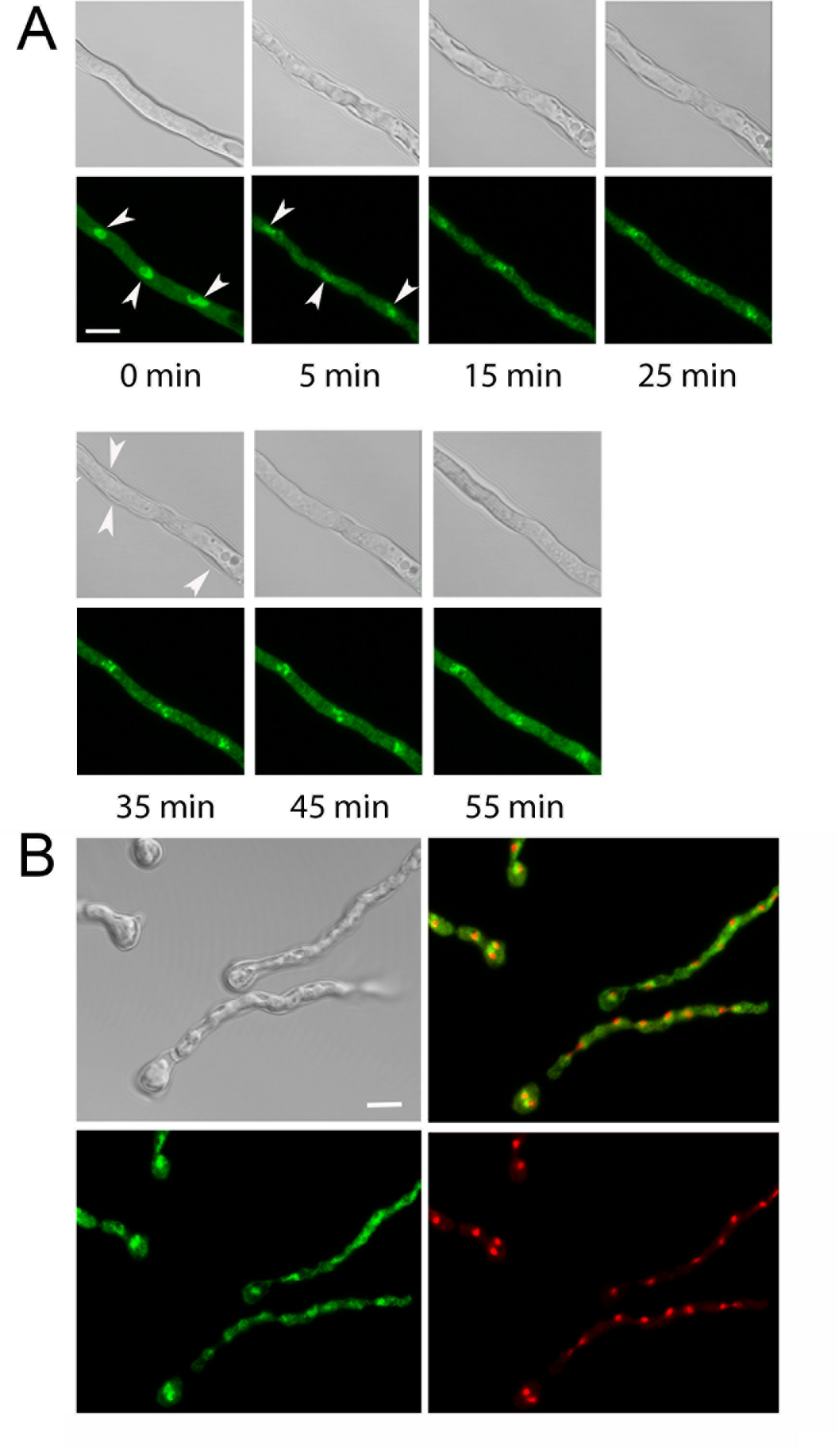
Redistribution of GFP-TcsC in hyphae that encountered a hyperosmotic shock. At t = 0 min, hyphae were transferred to a medium containing 1.2 M sorbitol. The presented images were taken at the indicated time points. The fluorescence images are maximum projections of confocal stacks. Panel A shows the distribution of GFP-TcsC after the shift to a hyperosmotic medium. Three nuclei are indicated by arrowheads in the images taken after 0 min and 5 min. The detachment of the cytoplasmic membrane from the cell wall is still visible 35 min after the hyperosmotic shock (indicated by arrowheads in the bright field image). The results of a similar experiment with a strain expressing GFP-TcsC- and fibrillarin-RFP are shown in panel B. All images of this panel were taken 35 min after the hyperosmotic shock. The distribution of GFP-TcsC is affected, whereas the localization pattern of fibrillarin-RFP remains undisturbed. The scale bars represent 5 µm and are valid for the respective set of panels.

TcsC enables hyphae to adapt to hyperosmotic stress (8); we, therefore, analyzed the localization of GFP-TcsC after a shift to a medium containing 1.2 M sorbitol. This treatment resulted in an immediate shrinkage of the cytoplasm that was evident as a retraction of the cellular membrane from the cell wall (Fig. 6A). GFP-TcsC showed a fast redistribution in response to high osmotic stress; the clearly defined nuclei disappeared, and individual nuclei became blurry and in some cases, their fluorescence nearly vanished. The cytoplasmic shrinkage was detectable until 35 to 40 min after the osmotic shock (Fig. 6A). At that time, the GFP-TcsC fluorescence in the nuclei was concentrated in small, dot-like structures that converged over time and a “normal” nuclear localization of GFP-TcsC was restored after approximately 55 min (Fig. 6A). Co-expression of TcsC-GFP and fibrillarin-RFP revealed that the morphology of the nucleoli was largely unaffected by the hyperosmotic shock (Fig. 6B).

Nanduri and Tartakoff (17) reported that a hyperosmotic shock induces a translocation of several nuclear proteins of *S. cerevisiae*, including Cbf5p, to the cytoplasm. This raised the possibility that the translocation of GFP-TcsC simply reflects a transient and unspecific release of nuclear proteins. To address this, we generated a GFP fusion of the *A. fumigatus* protein Afu5g05710 that shares 69.4% identical amino acids with *S. cerevisiae* Cbf5p. GFP-Afu5g05710 was targeted to the nucleus and remained there after a shift to a medium containing either 1.2 M sorbitol or 1 µg/mL fludioxonil. In hyphae that were exposed to 1.2 M sorbitol, the shape of the nuclei changed from a round to more drop-like morphology; treatment with fludioxonil had no impact on the morphology of the nuclei (Fig. S3). These data indicate that the overall structure of the nuclei is not dramatically changed by both treatments and that the observed redistribution of GFP-TcsC is specific for this protein.

**Fig 7:**
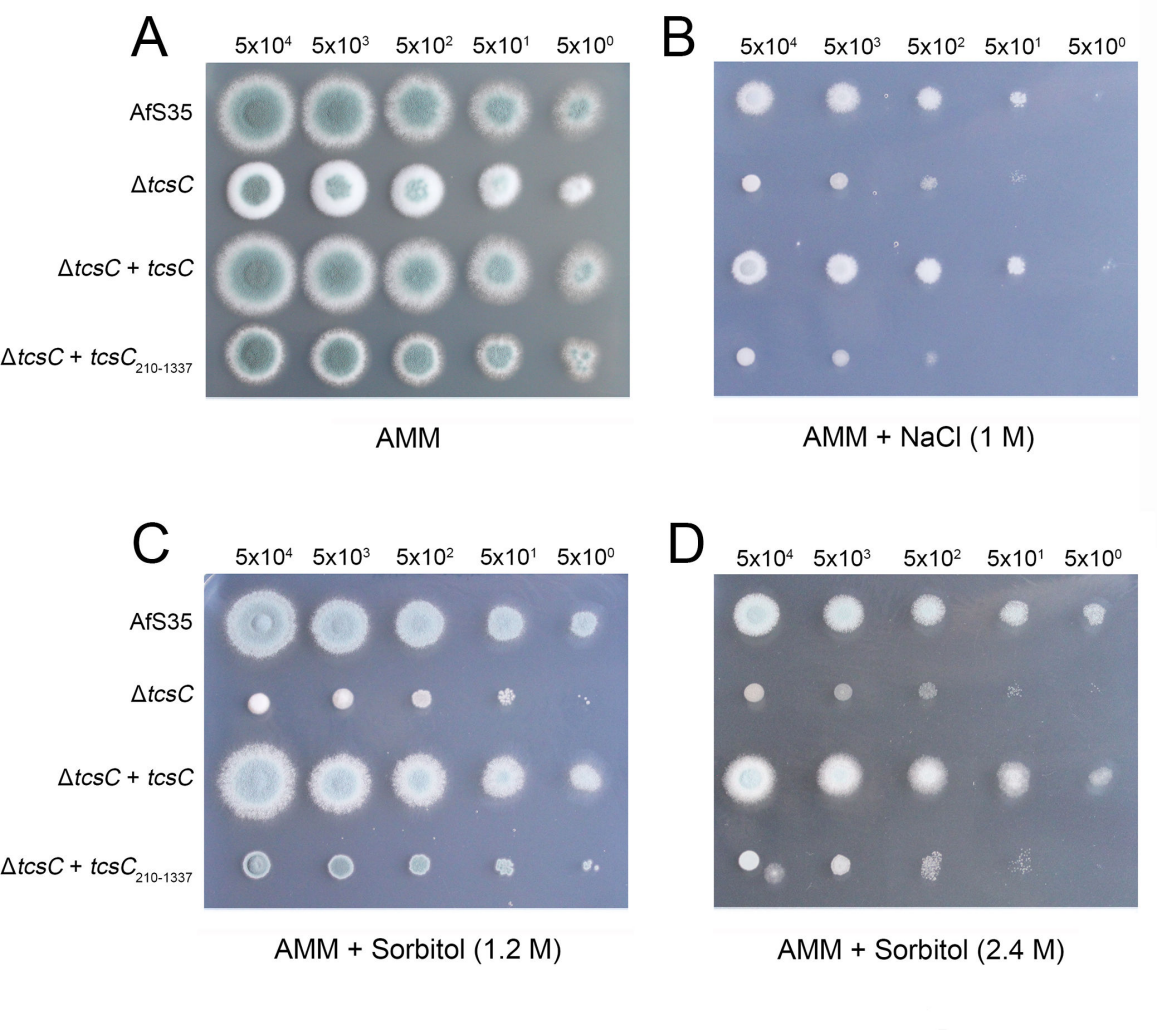
Functional characterization of TcsC_210–1337_. Drop dilution assays were performed with the indicated number of conidia per spot. The parental strain AfS35, its Δ*tcs*C mutant as well as the mutant expressing either full-length TcsC or TcsC_210–1337_ were grown at 37°C on plates with AMM for 48 h (panel A) and on AMM plates supplemented with either 1 M NaCl (panel B), 1.2 M sorbitol (panel C), or 2.4 M sorbitol (panel D) for 72 h.

We have previously reported that GFP-TcsC_210–1337_, which lacks the N-terminal sequence up to the first HAMP domain, shows a cytoplasmic localization and can mediate the antifungal activity of fludioxonil (16). We reinvestigated this strain and detected the GFP fusion protein in the cytoplasm, but not in the nuclei (Fig. S4). This demonstrates that the N-terminus is essential for the nuclear targeting. Expression of TcsC_210–1337_ in a Δ*tcs*C mutant led to a mixed phenotypic outcome: on *Aspergillus* minimal medium (AMM) plates, the slightly reduced colony size of the Δ*tcs*C mutant was not restored, but the TcsC_210–1337_-expressing strain lost the typical morphology of Δ*tcs*C colonies, namely, their white rim and their straight edge (Fig. 7A). On plates with hyperosmotic stress (1 M NaCl, 1.2 or 2.4 M sorbitol), the TcsC_210–1337_-expressing strain formed colonies that were much smaller than those of the wild type (Fig. 7B through D). This demonstrates that the N-terminus is essential for the physiological function of TcsC, namely, its ability to enable wild-type-like growth under hyperosmotic conditions.

**Fig 8:**
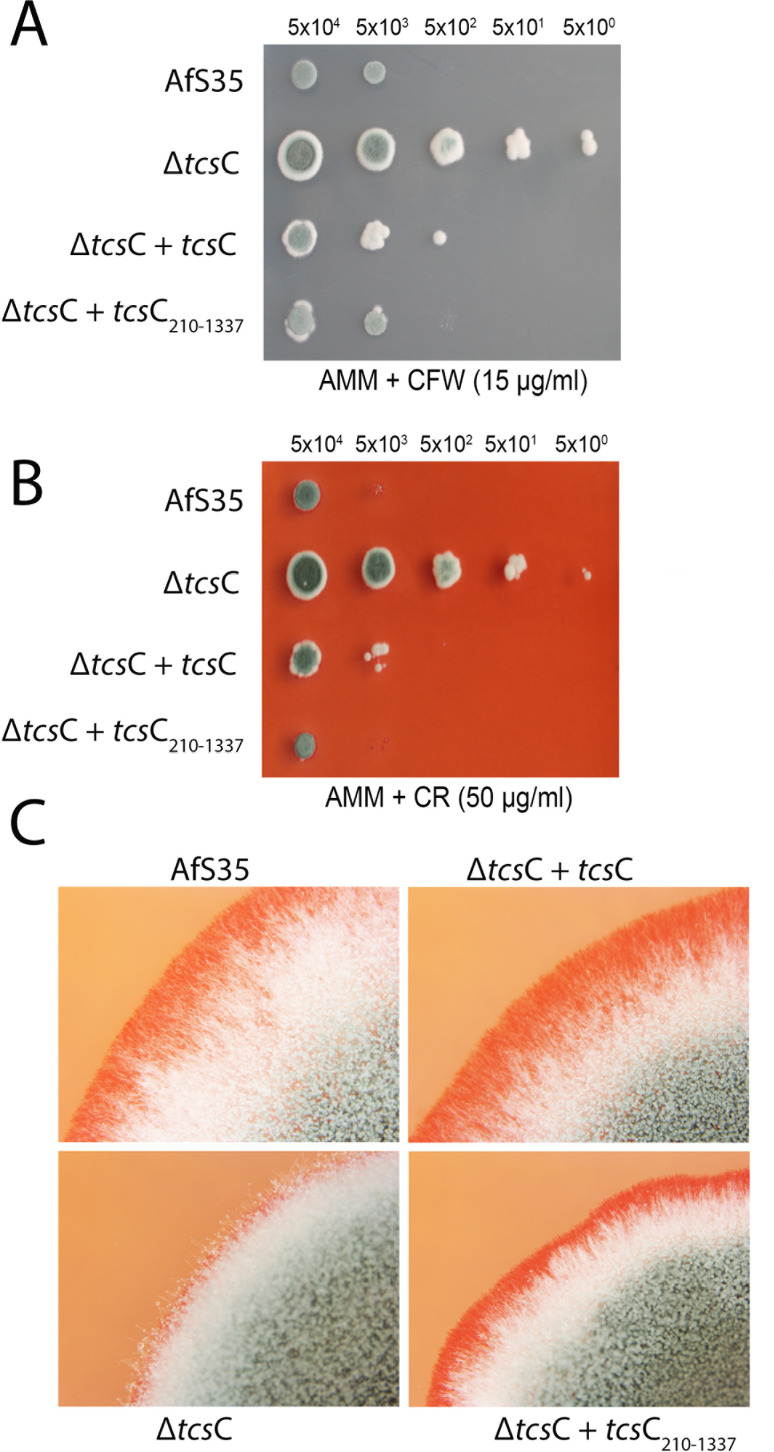
The enhanced resistance of the Δ*tcs*C mutant to cell walls stress is abolished after expression of TcsC_210–1337_. Drop dilution assays on plates containing 15 µg/mL CFW or 50 µg/mL CR are shown in panels A and B, respectively. The plates were inoculated with conidia of the indicated strains and incubated for 48 h at 37°C. The numbers of conidia per spot are indicated. Microscopic images showing close-ups of the edge of colonies grown on CR-containing plates are shown in panel C.

Another striking feature of the Δ*tcs*C mutant is its enhanced resistance to the cell wall stressors CFW and CR, a phenotype that disappeared after the expression of TcsC_210–1337_ (Fig. 8A and B, respectively). On CR-containing plates, colonies of the wild type, the complemented Δ*tcs*C mutant, and the mutant expressing TcsC_210–1337_ had a pronounced red periphery, a feature that was less evident for the Δ*tcs*C colonies (Fig. 8C). This indicates that the presence of TcsC or TcsC_210–1337_ results in an increased sensitivity to these stressors. The different CR binding to the colonies suggests that TcsC and TcsC_210–1337_ modulate the cell wall architecture and thereby generate additional binding sites for CR. We have recently shown that the cell walls of the Δ*tcs*C mutant and the wild type differ in their composition, e.g., the amount of alpha-1,3-glucan is much lower in the mutant (18), which implies that, even in the absence of stress, TcsC has an impact on the cell wall architecture.

**Fig 9:**
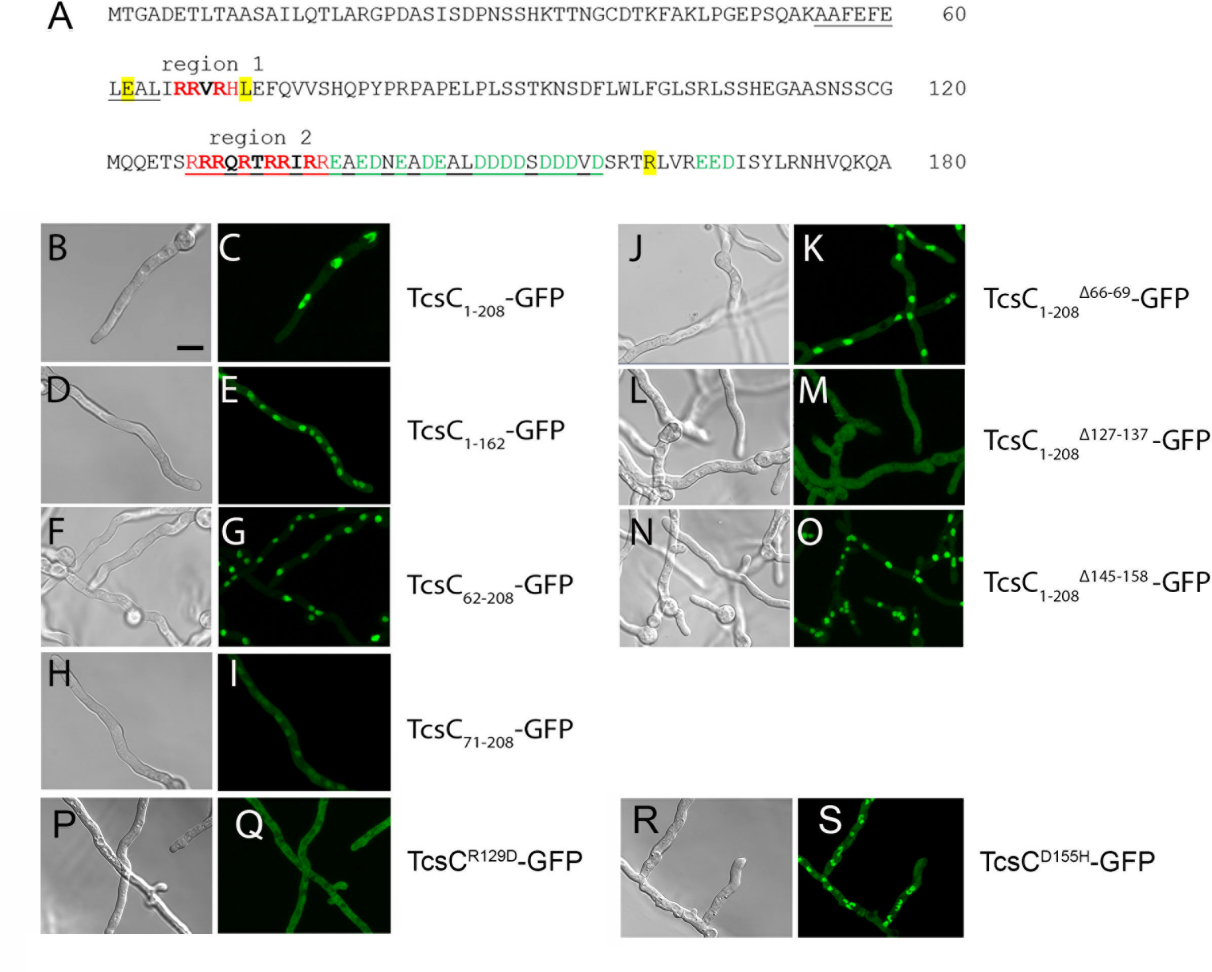
Analysis of the N-terminal part of TcsC. The N-terminal 208 aa of TcsC are depicted in panel A. The predicted nuclear localization sequences (NLS) are indicated in bold, and clusters of basic and acid residues are written in red or green letters, respectively. Positions that mark the boundaries of further truncated fusion proteins are highlighted in yellow. Recruitment of TcsC_1–208_-GFP, TcsC_62–208_-GFP, TcsC_1–162_-GFP, TcsC_1–208_^Δ145–158^-GFP, and TcsC_1–208_^D155H^-GFP to the nucleus is shown in panels C, E, G, O, and S, respectively. The corresponding bright field images are depicted in panels B, D, F, N, and R. A reduced nuclear targeting of TcsC_71–208_-GFP, which lacks region 1, is shown in panel I, and the corresponding bright field image in panel H. The abolished nuclear recruitment of TcsC_1–208_^Δ127–137^-GFP and TcsC_1–208_^R129D^-GFP is evident in M and Q, respectively. The corresponding bright field images are shown in L and P. The bar in B represents 5 µm and is valid for all images.

In conclusion, the data presented so far demonstrate that the N-terminal 209 aa of TcsC are required for the nuclear localization and the response to hyperosmotic stress, but not for the antifungal effect of fludioxonil or a wild-type-like sensitivity to cell wall stressors. In the next step, we constructed a TcsC_1–208_-GFP fusion, which lacks all HAMP domains and the complete signaling module. After expression in AfS35, TcsC_1–208_-GFP was efficiently targeted to the nucleus (Fig. 9C).

Analysis of the N-terminal 208 aa for putative domains or motifs revealed only two regions of low complexity corresponding to positions 55–64 and 127–158 (Fig. 1B and 9A). However, we identified three charged motifs (Fig. 9A): an acidic region (aa 138–158, indicated in green) as well as a shorter and a longer basic region (region 1: aa 66–69 and region 2: aa 127–137; indicated in red). The basic regions harbor three sequences that resemble classical monopartite NLS (consensus K(K/R) X(K/R)): _66_RRVR_69_, _128_RRQR_131_, and _133_RRIR_136_ (Fig. 9A, indicated in bold) and TcsC_1–208_ alone is predicted to be a nuclear protein (PSORT II; reliability of 94.1). We generated two additional fusion proteins, TcsC_1–162_-GFP and TcsC_62–208_-GFP harboring all charged regions, but each lacking one of the flanking sequences. Both fusions showed a nuclear localization (Fig. 9E and G), but a further truncation to TcsC_71–208_ resulted in a much weaker nuclear fluorescence (Fig. 9I), which suggests that the RRVR motif of region 1 is not essential but contributes to an efficient targeting.

A deletion of region 2 in TcsC_1–208_^Δ127–137^-GFP abolished the nuclear targeting (Fig. 8M), whereas TcsC_1–208_^Δ145–158^-GFP, which lacks the acidic region, showed a clear nuclear localization (Fig. 9O). Loss of region 1 in TcsC_1–208_^Δ66–69^-GFP had no obvious impact on the nuclear targeting, indicating that this motif is not essential (Fig. 9K). In conclusion, these data demonstrate that nuclear targeting depends primarily on the basic region 2.

**Fig 10:**
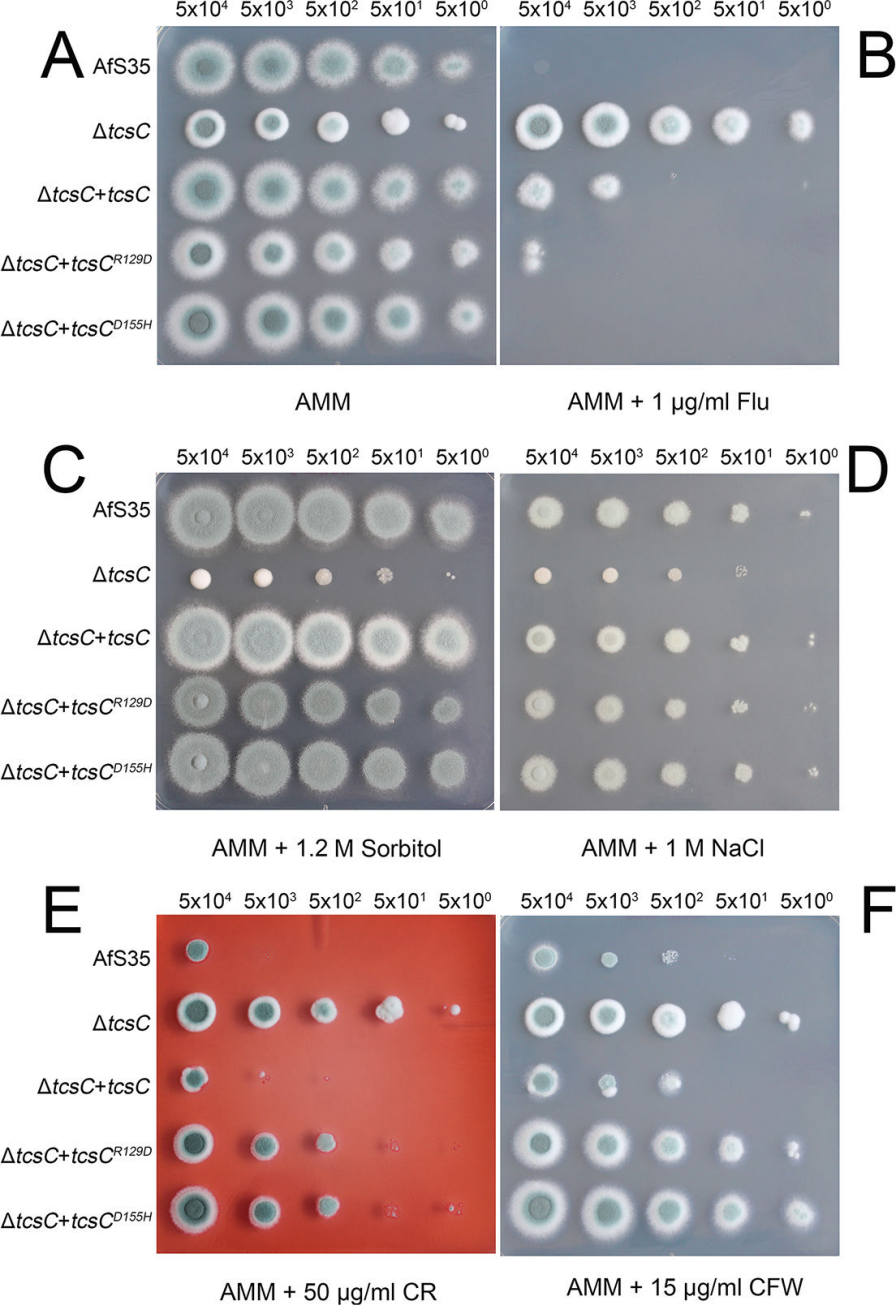
Phenotypic analysis of strains Δ*tcs*C + *tcs*C^R129D^ and Δ*tcs*C + *tcs*C^D155H^. The two strains under investigation and the control strains AfS35 (wild type), Δ*tcs*C, and Δ*tcs*C + *tcs*C were grown at 37°C on the indicated media. The numbers of conidia per spot and the concentrations of the supplements are indicated.

To further examine the functional importance of the charged regions, one amino acid was mutated in each motif; we introduced an acidic residue in the basic motif and vice versa. After expression in the *A. fumigatus* wild type or the Δ*tcs*C mutant, GFP-TcsC^R129D^ was localized in the cytoplasm, whereas GFP-TcsC^D155H^ was targeted to the nucleus (Fig. 9Q and S, respectively, and data not shown). This demonstrates that the R129D exchange alone is sufficient to prevent a nuclear targeting. Although we observed no defect in the nuclear localization of TcsC^D155H^, it is remarkable that the morphology of this strain was striking with hyphae showing an irregular morphology (Fig. 9R). Phenotypic testing of ∆*tcs*C strains expressing either TcsC, TcsC^R129D^, or TcsC^D155H^ revealed that all of them resembled the wild type in their sensitivity to fludioxonil and their ability to adapt to hyperosmotic stress (Fig. 10B through D, respectively). Hence, both mutated forms of TcsC were functional, although they reside in different cellular compartments under resting conditions. On AMM, the colonies of the mutant expressing TcsC^R129D^ were slightly smaller and had a more pronounced white rim compared to the wild type and were more similar to colonies of the Δ*tcs*C mutant. On plates containing the cell wall stressors CR or CFW, expression of both mutated TcsC proteins resulted in an increased resistance as is typical for the Δ*tcs*C mutant (Fig. 10E and F). Hence, TcsC has an impact on the resistance level to CFW or CR in the wild type, but not in strains expressing either TcsC^R129D^ or TcsC^D155H^.

**Fig 11:**
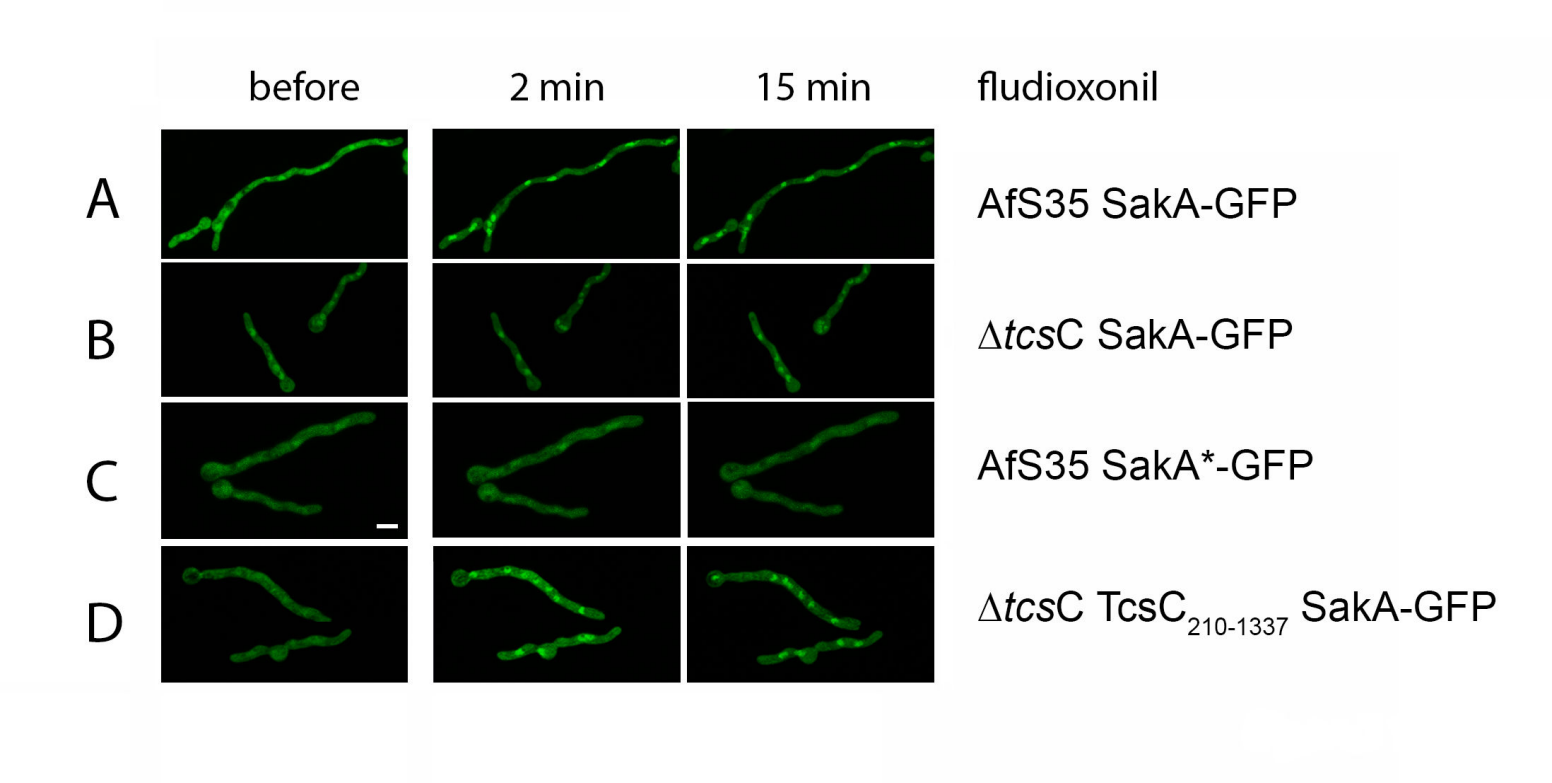
Impact of fludioxonil on the localization of SakA-GFP. Images were taken before and at the indicated time points after the addition of fludioxonil (1 µg/mL). GFP images of the wild-type strain AfS35 and its Δ*tcs*C mutant are shown in panels A and B, respectively. SakA*-GFP indicates a mutation of the putative phosphorylation site of SakA. In the wild type, SakA*-GFP showed no translocation in response to fludioxonil (panel C). The localization of SakA-GFP in Δ*tcs*C expressing TcsC_210–1335_ is shown in panel D. The bar in panel C represents 5 µm and is valid for all images.

The data presented so far indicate that activation of TcsC causes its redistribution within the cell. TcsC executes its physiological and antifungal activity via the response regulators Skn7 and SakA (11). Skn7 is a nuclear protein, both in its activated or non-activated form, whereas SakA translocates from the cytoplasm to the nucleus upon activation. In the AfS35 wild type, SakA-GFP translocated to the nucleus within 2 min after the addition of fludioxonil (Fig. 11A). In the ∆*tcs*C mutant, GFP-SakA showed no translocation in response to fludioxonil; instead, we observed a weak nuclear enrichment of the fusion protein at all time points and in the presence and absence of fludioxonil (Fig. 11B). This demonstrates that SakA is activated in a TcsC-dependent manner and this activation occurs before a redistribution of TcsC from the nucleus to the cytoplasm is evident. In *S. cerevisiae*, Hog1p becomes activated by phosphorylation (19), and the corresponding site is well-conserved in SakA. We have mutated the residues TGY at positions 171 to 173 of SakA to AGA. The resulting GFP fusion protein, GFP-SakA*, showed no nuclear translocation in response to fludioxonil and was hardly detectable in the nuclei of resting hyphae (Fig. 11C). This demonstrates that the translocation of SakA to the nucleus is triggered by its phosphorylation at residues 171 and/or 173. The fludioxonil-induced translocation of SakA-GFP to the nucleus was also detectable in ∆*tcs*C strains expressing either TcsC_210–1337_ (Fig. 11D) or TcsC^R129D^ (data not shown) indicating that both proteins can activate the MAP kinase cascade of the HOG pathway.

## DISCUSSION

We identified TcsC as the first group III HHK that is recruited to the nucleus. This targeting occurs no matter whether TcsC is fused to the N- or C-terminus of GFP. Proteins that are smaller than 60 kDa can freely diffuse through the nuclear pore (20, 21), but TcsC with a molecular weight of approximately 150 kDa requires the nuclear import machinery to pass through the nuclear pore. Remarkably, GFP-TcsC is excluded from the nucleoli, a membrane-less organelle within the nucleus where ribosome biogenesis takes place. From the different GFP-TcsC fusion proteins that were analyzed in this study, only some show this exclusion from the nucleolus.

The information for the nuclear targeting resides in the N-terminal 208 aa of TcsC. A fusion protein comprising this part of TcsC is small enough to travel freely through the nuclear pore. Since nucleoli are not separated by membranes from the rest of the nucleus the exclusion of TcsC_1–208_-GFP from the nucleolus indicates that these truncated TcsC moieties bind to yet unknown molecules or structures, that are present in the nucleus, but not in the nucleolus.

**Fig 12:**
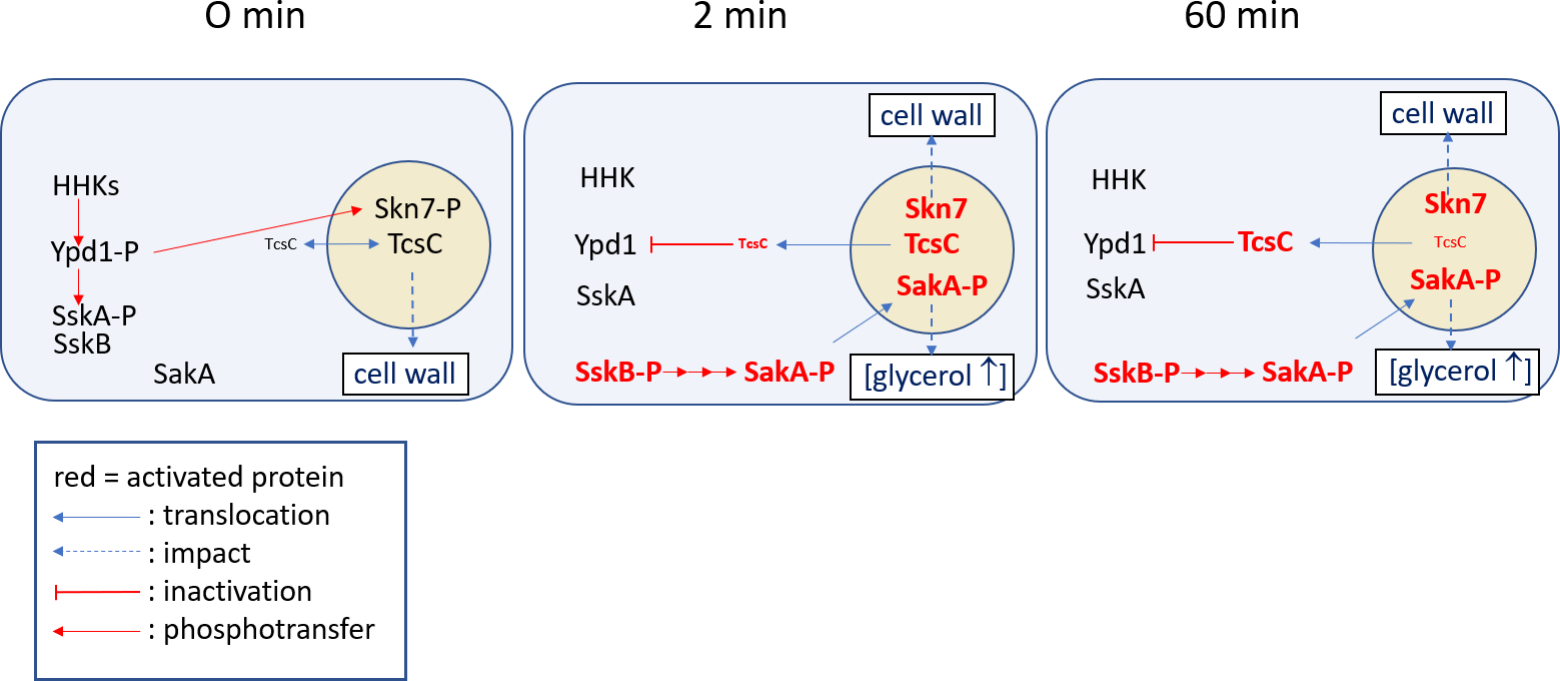
Model of events that are triggered by fludioxonil at an early (2 min) and later time point (60 min). The names of activated proteins are written in red. The size of the letters indicates differences in the abundance of TcsC in the different cellular compartments. The dimer formation between phospho-SskA and SskB results in inactivation of the MAP kinase cascade from SskB to SakA.

Bleaching experiments indicate a slow shuttling of full-length GFP-TcsC molecules between the cytoplasm and the nucleus. Activation of TcsC by fludioxonil results in cytoplasmic localization of the vast majority of the GFP-TcsC proteins, which requires approximately 2 h. Removal of fludioxonil induced a re-localization of the nuclei in a roughly similar time course. Hence, the kinetics of the translocation in either direction are slow. We initially assumed that the translocation of TcsC indicates an activation, but this concept is challenged by two observations: the TcsC-dependent phosphorylation and translocation of SakA occurs already 2 min after the addition of fludioxonil (this study; 8) and RNAseq data indicate a strong and TcsC-dependent transcriptional response 1 h after the addition of fludioxonil (18). The migration of TcsC from the nucleus to the cytoplasm represents therefore a late event in the response to fludioxonil. A model that depicts the situation before as well as 2 and 60 min after the addition of fludioxonil is shown in Fig. 12. According to this model, fludioxonil activates TcsC immediately, which is in line with the fact that fludioxonil induces an immediate growth arrest of *A. fumigatus* hyphae (9). An activation of SakA requires TcsC and is evident after 2 min in the presence of fludioxonil, a time point when only low amounts of TcsC are present in the cytoplasm. Activation of SakA results in an elevated osmotic pressure in the cytoplasm. Other, largely Skn7-dependent processes run in parallel and weaken the cell wall, which paves the way for the dramatic morphological changes that are characteristic of fludioxonil-treated hyphae. We hypothesize that activated TcsC proteins are retained in the cytoplasm, which then slowly depletes TcsC from the nucleus (Fig. 12). The slow kinetics of this process correlates well to the gradual turn-over of TcsC in the nuclei of non-stressed cells.

The TcsC molecule can be divided into an N-terminal sensing molecule (aa 1–750) and a C-terminal signaling module (aa 751–1337) (16). According to the concept of Bahn (22), the signaling module becomes activated and delivers phosphoryl groups to the downstream HPt protein Ypd1. In *A. fumigatus*, Ypd1 is present in the whole nucleo-cytoplasm and shuttles between both compartments. Ypd1 translocates much faster than TcsC (12), which is likely due to its much smaller size.

TcsC interacts with Ypd1 to modulate the activities of the two response regulators SakA and Skn7. For the antifungal activity of fludioxonil, Skn7 is more important than SakA (11). Under resting conditions, SakA is a cytosolic protein that translocates to the nucleus upon activation, whereas Skn7 resides in the nucleus both, in the presence and absence of stress (12). Due to the ability of Ypd1 to shuttle freely between the nucleus and the cytoplasm, TcsC can activate both response regulators independent of its localization.

In response to hyperosmotic stress, hyphal cells show a fast shrinkage of the cytoplasm that entails a partial detachment of the cytoplasmic membrane from the cell wall. Concomitantly, a high percentage of GFP-TcsC proteins migrated from the nuclei to the cytoplasm. Approximately 40 min after the hyperosmotic shock, the normal cellular morphology was restored. In this adaptation phase, GFP-TcsC became enriched in distinct spots within the nuclei. A similar enrichment in punctuated structures after exposure to hyperosmotic stress was reported for the TcsC ortholog Nik1 of *C. guilliermondii*, but in the yeast, these spots were not restricted to the nucleus (15). The nature of the TcsC-positive nuclear structures and their relevance for the adaptive response to hyperosmotic stress is still an open issue. Co-expression with the nucleoli-specific protein fibrillarin indicates that the TcsC-positive spots and the nucleoli are different cellular structures. There are similarities between the TcsC-containing spots and nuclear stress bodies and stress granules in mammalian cells (23, 24) that may help to tackle this point in the future. After the hyperosmotic shock, the TcsC-positive spots enlarged and merged to finally restore a normal nuclear enrichment of TcsC. The overall structure of the nuclei was not affected by the hyperosmotic shock. In conclusion, these data demonstrate that both, fludioxonil and hyperosmotic stress cause a re-localization of GFP-TcsC that is reversed after removal of the antifungal or as soon as a new osmotic homeostasis is established.

A truncated TcsC lacking the N-terminal 209 aa resides in the cytoplasm and is excluded from the nucleus. This together with the nuclear targeting of TcsC_1–208_-GFP demonstrates that the N-terminus of TcsC is both, essential and sufficient for the recruitment to and the retention in the nucleus. An *A. fumigatus* strain expressing only TcsC_210–1337_ is unable to cope with hyperosmotic stress but is sensitive to fludioxonil. Hence, the N-terminus is required for the nuclear targeting and the physiological function of TcsC, but not for its interaction with fludioxonil.

Proteins are guided to the nucleus by motifs that are recognized by the nuclear import machinery (20), e.g., the classical monopartite NLS with the consensus sequence K(K/R)X(K/R). Three very similar motifs exist in the N-terminus of TcsC, one in region 1 (RRVR; aa 66–69) and two adjacent motifs in region 2 (RRQR and RRIR; aa 128–136). Experiments with GFP fusions harboring the N-terminus of TcsC, but lacking either region 1 or 2 indicate that only region 2 is essential for the nuclear targeting.

To analyze this in more detail, we expressed TcsC^R129D^ in the genetic background of a ∆*tcs*C mutant and analyzed its biological activities. TcsC^R129D^ is no longer targeted to the nucleus, but this mis-localization was not associated with a functional defect, since TcsC^R129D^ enabled wild-type-like responses to fludioxonil and hyperosmotic stress. In contrast, the loss of the N-terminal 208 amino acids renders TcsC unable to respond to hyperosmotic stress, which indicates that this part of the protein is essential for this adaptive response.

The distinct morphology of Δ*tcs*C mutant colonies (8) indicates that TcsC fulfills certain functions in non-stressed hyphae when the protein resides in the nucleus. The finding that the colonies of the TcsC^R129D^-expressing strain resembled those of the Δ*tcs*C mutant provides a first hint that the nuclear localization of TcsC may be important for these functions. Another observation that points in this direction is that TcsC^R129D^- or TcsC^D155H^-expressing strains showed a high level of resistance to the cell wall stressors CFW and CR, similar to the Δ*tcs*C mutant. This resistance indicates that TcsC modulates the cell wall in a way that renders hyphae more sensitive to CFW and CR. The high resistance levels of the TcsC^R129D^- and the TcsC^D155H^-expressing strains suggest that this activity requires recruitment of TcsC to the nucleus and depends on the acidic motif in the N-terminus of TcsC, respectively. The irregular morphology of hyphae that express TcsC^D155H^ provides further support for this concept.

In summary, this study has identified the N-terminus of TcsC as a functionally important part of the protein. The N-terminus guides the protein to the nucleus, but this distinct localization is not required for its function in the adaptive stress response. Hence, our data suggest that TcsC switches between two activities: in the absence of stress, TcsC influences the cell wall composition and this requires its nuclear localization; after activation by hyperosmotic stress or fludioxonil, TcsC activates the HOG pathway. These distinct activities may reflect a switch in the enzymatic activity of TcsC as it was previously reported for the group III HHK Drk1 of *Blastomyces dermatitidis* that was shown to switch from a kinase activity under resting conditions to a phosphatase activity in the activated state (25). Our data demonstrate a well-orchestrated spatiotemporal dynamic of TcsC, which represents another element in the complex architecture of the multistep phosphorelay. Further research is necessary to analyze the functional significance of these entangled processes in more detail.

## MATERIALS AND METHODS

### Strains

The strains that were used in this study are summarized in Table S1. If not stated otherwise the fungal strains were grown on plates containing AMM with or without further supplements as indicated in the text. The recipe of AMM and the procedure used to isolate conidia were described previously (26).

### Generation of mutant strains

Strain AfS35, which lacks a functional non-homologous end-joining system, was used to generate all mutant strains (27). For cloning experiments, all PCR reactions were performed with the Q5 High Fidelity DNA Polymerase (New England Biolabs). All oligonucleotides are listed in Table S2.

The plasmids pSK379, pSK379-RFP-Phleo, and pSK379-GFP have been described previously (12, 28). Suitable PCR products were cloned into the PmeI or EcoRV site of the target vector and after transformation, the fusion proteins were expressed from the vector-derived *gpd*A promoter. To introduce point mutations or deletions, we used a suitable plasmid and mutations were introduced with the Q5 Site-Directed Mutagenesis Kit (New England Biolabs). The respective oligonucleotides were designed using the NEBaseChanger software (https://nebasechanger.neb.com/). All constructs were verified by sequencing and the plasmids were then introduced into protoplasts of the appropriate target strain.

To obtain a strain with tagged nuclei, the C-terminal part of the *stu*A gene of *A. fumigatus* (Afu2g07900) was amplified using oligonucleotides StuA in EcoRV-FOR and StuA in EcoRV-REV (29). The resulting fragment of 471 bp was cloned in the proper orientation into the EcoRV site of pSK379-RFP-hygro and the plasmid was subsequently introduced into a GFP-TcsC expressing AfS35 strain. After transformation, strains derived from colonies with the expected resistance were verified by PCR and in the case of mutants that carried substitutions or short deletions also by DNA sequencing. Strains expressing GFP or RFP construct were furthermore analyzed by fluorescent microscopy.

### Sequence analysis

Sequences were compared using Clustal Omega (https://www.ebi.ac.uk/Tools/msa/clustalo/). The intracellular localization of TcsC was predicted using WoLF PSORT (https://wolfpsort.hgc.jp) and pSORT II (https://psort.hgc.jp).

### Microscopy

To determine the spatial distribution of GFP fusion proteins, we grew the respective strains in AMM in cell culture multi-well chambers or µ-slides (IBIDI, Martinsried, Germany). Viable hyphae were then analyzed using a confocal laser scanning microscope Zeiss LSM880 with an attached climate chamber that was adjusted to 37°C. For bleaching experiments, a part of the sample (indicated as a boxed area in the respective image) was bleached with 488 nm laser light until the GFP signals had disappeared. Videos were taken using the ZEN black software (Zeiss, Germany).

Microscopic images of colonies grown on agar plates were taken using a Leica DM5000B microscope with an attached DFC3000G camera.

### Phenotypic tests

For drop dilution assays, freshly isolated conidia were counted using a Neubauer-improved chamber, and a series of 10-fold dilutions were generated starting with 2 × 10^7^ conidia per milliliter. The indicated number of conidia were spotted in droplets of 2.5 µL onto AMM plates with or without supplements. If not stated otherwise, plates were incubated for 48 h at 37°C, and only sorbitol- and NaCl-containing plates were incubated for 72 h. High-resolution images of fungal colonies grown on CR-containing plates were taken using a Leica M205C microscope and an attached Leica MC170 HD camera (Leica Microsystems).

To determine whether the translocation of TcsC is an active process, GFP-TcsC- and fibrillarin-RFP expressing short hyphae were pre-incubated with 200 µM CCCP; fludioxonil was then added to the medium at a final concentration of 1 µg/mL and the samples were analyzed by live cell microscopy.

## SUPPLEMENTAL MATERIAL

Fig. S1 mbio.01184-24-s0001.tif Targeting of TcsC to the nucleus.

Fig. S2 mbio.01184-24-s0002.tif The kinase domain of TcsC is required for the translocation.

Fig. S3 mbio.01184-24-s0003.tif Localization GFP-Afu5g05710.

Fig. S4 mbio.01184-24-s0004.tif GFP-TcsC_210-1337_ is not recruited to the nucleus.

Legends mbio.01184-24-s0005.docx Legends for supplemental figures.

Table S1 mbio.01184-24-s0006.docx Strains used in this study.

Table S2 mbio.01184-24-s0007.docx Oligonucleotides used in this study.

ASM does not own the copyrights to Supplemental Material that may be linked to, or accessed through, an article. The authors have granted ASM a non-exclusive, world-wide license to publish the Supplemental Material files. Please contact the corresponding author directly for reuse.

## References

[1] Defosse TA, Sharma A, Mondal AK, Dugé de Bernonville T, Latgé J-P, Calderone R, Giglioli-Guivarc’h N, Courdavault V, Clastre M, Papon N. 2015. Hybrid histidine kinases in pathogenic fungi. Mol Microbiol 95:914–924. doi:10.1111/mmi.12911 25560420

[2] Inoue K, Sasaki J, Spudich JL, Terazima M. 2008. Signal transmission through the HtrII transducer alters the interaction of two alpha-helices in the HAMP domain. J Mol Biol 376:963–970. doi:10.1016/j.jmb.2007.12.026 18199454

[3] Airola MV, Watts KJ, Bilwes AM, Crane BR. 2010. Structure of concatenated HAMP domains provides a mechanism for signal transduction. Structure 18:436–448. doi:10.1016/j.str.2010.01.013 20399181 PMC2892831

[4] Hérivaux A, So YS, Gastebois A, Latgé JP, Bouchara JP, Bahn YS, Papon N. 2016. Major sensing proteins in pathogenic fungi: the hybrid Histidine kinase family. PLoS Pathog 12:e1005683. doi:10.1371/journal.ppat.1005683 27467512 PMC4965123

[5] Catlett NL, Yoder OC, Turgeon BG. 2003. Whole-genome analysis of two-component signal transduction genes in fungal pathogens. Eukaryot Cell 2:1151–1161. doi:10.1128/EC.2.6.1151-1161.2003 14665450 PMC326637

[6] Brewster JL, Gustin MC. 2014. Hog1: 20 years of discovery and impact. Sci Signal 7:re7. doi:10.1126/scisignal.2005458 25227612

[7] Kojima K, Takano Y, Yoshimi A, Tanaka C, Kikuchi T, Okuno T. 2004. Fungicide activity through activation of a fungal signalling pathway. Mol Microbiol 53:1785–1796. doi:10.1111/j.1365-2958.2004.04244.x 15341655

[8] McCormick A, Jacobsen ID, Broniszewska M, Beck J, Heesemann J, Ebel F. 2012. The two-component sensor kinase TcsC and its role in stress resistance of the human-pathogenic mold Aspergillus fumigatus. PLoS One 7:e38262. doi:10.1371/journal.pone.0038262 22675534 PMC3366943

[9] Wiedemann A, Spadinger A, Löwe A, Seeger A, Ebel F. 2016. Agents that activate the high osmolarity glycerol pathway as a means to combat pathogenic molds. Int J Med Microbiol 306:642–651. doi:10.1016/j.ijmm.2016.09.005 27713026

[10] Shor E, Chauhan N. 2015. A case for two-component signaling systems as antifungal drug targets. PLoS Pathog 11:e1004632. doi:10.1371/journal.ppat.1004632 25723524 PMC4344368

[11] Schruefer S, Böhmer I, Dichtl K, Spadinger A, Kleinemeier C, Ebel F. 2021. The response regulator Skn7 of Aspergillus fumigatus is essential for the antifungal effect of fludioxonil. Sci Rep 11:5317. doi:10.1038/s41598-021-84740-6 33674651 PMC7935864

[12] Schruefer S, Spadinger A, Kleinemeier C, Schmid L, Ebel F. 2021. Ypd1 is an essential protein of the major fungal pathogen Aspergillus fumigatus and a key element in the phosphorelay that is targeted by the antifungal drug fludioxonil. Front Fungal Biol 2:756990. doi:10.3389/ffunb.2021.756990 37744118 PMC10512271

[13] Posas F, Wurgler-Murphy SM, Maeda T, Witten EA, Thai TC, Saito H. 1996. Yeast HOG1 MAP kinase cascade is regulated by a multistep phosphorelay mechanism in the SLN1-YPD1-SSK1 "two-component" osmosensor. Cell 86:865–875. doi:10.1016/s0092-8674(00)80162-2 8808622

[14] Meena N, Kaur H, Mondal AK. 2010. Interactions among HAMP domain repeats act as an osmosensing molecular switch in group III hybrid histidine kinases from fungi. J Biol Chem 285:12121–12132. doi:10.1074/jbc.M109.075721 20164185 PMC2852951

[15] Foureau E, Clastre M, Montoya EJO, Besseau S, Oudin A, Glévarec G, Simkin AJ, Crèche J, Atehortùa L, Giglioli-Guivarc’h N, Courdavault V, Papon N. 2014. Subcellular localization of the histidine kinase receptors Sln1p, Nik1p and Chk1p in the yeast CTG clade species Candida guilliermondii. Fungal Genet Biol 65:25–36. doi:10.1016/j.fgb.2014.01.007 24518307

[16] Spadinger A, Ebel F. 2017. Molecular characterization of Aspergillus fumigatus TcsC, a characteristic type III hybrid histidine kinase of filamentous fungi harboring six HAMP domains. Int J Med Microbiol 307:200–208. doi:10.1016/j.ijmm.2017.05.002 28527583

[17] Nanduri J, Tartakoff AM. 2001. Perturbation of the nucleus: a novel Hog1p-independent, Pkc1p-dependent consequence of hypertonic shock in yeast. Mol Biol Cell 12:1835–1841. doi:10.1091/mbc.12.6.1835 11408589 PMC37345

[18] Schruefer S, Pschibul A, Wong SSW, Sae-Ong T, Wolf T, Schäuble S, Panagiotou G, Brakhage AA, Aimanianda V, Kniemeyer O, Ebel F. 2023. Distinct transcriptional responses to fludioxonil in Aspergillus fumigatus and its ΔtcsC and Δskn7 mutants reveal a crucial role for Skn7 in the cell wall reorganizations triggered by this antifungal. BMC Genomics 24:684. doi:10.1186/s12864-023-09777-5 37964194 PMC10647056

[19] Ferrigno P, Posas F, Koepp D, Saito H, Silver PA. 1998. Regulated nucleo/cytoplasmic exchange of HOG1 MAPK requires the importin beta homologs NMD5 and XPO1. EMBO J 17:5606–5614. doi:10.1093/emboj/17.19.5606 9755161 PMC1170889

[20] Lusk CP, Blobel G, King MC. 2007. Highway to the inner nuclear membrane: rules for the road. Nat Rev Mol Cell Biol 8:414–420. doi:10.1038/nrm2165 17440484

[21] Wang R, Brattain MG. 2007. The maximal size of protein to diffuse through the nuclear pore is larger than 60kDa. FEBS Lett 581:3164–3170. doi:10.1016/j.febslet.2007.05.082 17588566 PMC4064367

[22] Bahn YS. 2008. Master and commander in fungal pathogens: the two-component system and the HOG signaling pathway. Eukaryot Cell 7:2017–2036. doi:10.1128/EC.00323-08 18952900 PMC2593196

[23] van Leeuwen W, Rabouille C. 2019. Cellular stress leads to the formation of membraneless stress assemblies in eukaryotic cells. Traffic 20:623–638. doi:10.1111/tra.12669 31152627 PMC6771618

[24] Hirose T, Ninomiya K, Nakagawa S, Yamazaki T. 2023. A guide to membraneless organelles and their various roles in gene regulation. Nat Rev Mol Cell Biol 24:288–304. doi:10.1038/s41580-022-00558-8 36424481

[25] Lawry SM, Tebbets B, Kean I, Stewart D, Hetelle J, Klein BS. 2017. Fludioxonil induces Drk1, a fungal group III hybrid histidine kinase, to dephosphorylate its downstream target, Ypd1. Antimicrob Agents Chemother 61:e01414-16. doi:10.1128/AAC.01414-16 27872062 PMC5278731

[26] Rohde M, Schwienbacher M, Nikolaus T, Heesemann J, Ebel F. 2002. Detection of early phase specific surface appendages during germination of Aspergillus fumigatus conidia. FEMS Microbiol Lett 206:99–105. doi:10.1111/j.1574-6968.2002.tb10993.x 11786264

[27] Krappmann S, Sasse C, Braus GH. 2006. Gene targeting in Aspergillus fumigatus by homologous recombination is facilitated in a nonhomologous end- joining-deficient genetic background. Eukaryot Cell 5:212–215. doi:10.1128/EC.5.1.212-215.2006 16400185 PMC1360265

[28] Beck J, Ebel F. 2013. Characterization of the major Woronin body protein HexA of the human pathogenic mold Aspergillus fumigatus. Int J Med Microbiol 303:90–97. doi:10.1016/j.ijmm.2012.11.005 23332467

[29] Suelmann R, Sievers N, Fischer R. 1997. Nuclear traffic in fungal hyphae: in vivo study of nuclear migration and positioning in Aspergillus nidulans. Mol Microbiol 25:757–769. doi:10.1046/j.1365-2958.1997.5131873.x 9379904

