# Supplementary figures and images for: The N-terminus of the Aspergillus fumigatus group III hybrid histidine kinase TcsC is essential for its physiological activity and targets the protein to the nucleus
Source: mBio. 2024 Jun 4;15(7):e01184-24. doi: 10.1128/mbio.01184-24 (PMC11253588; doi:10.1128/mbio.01184-24)

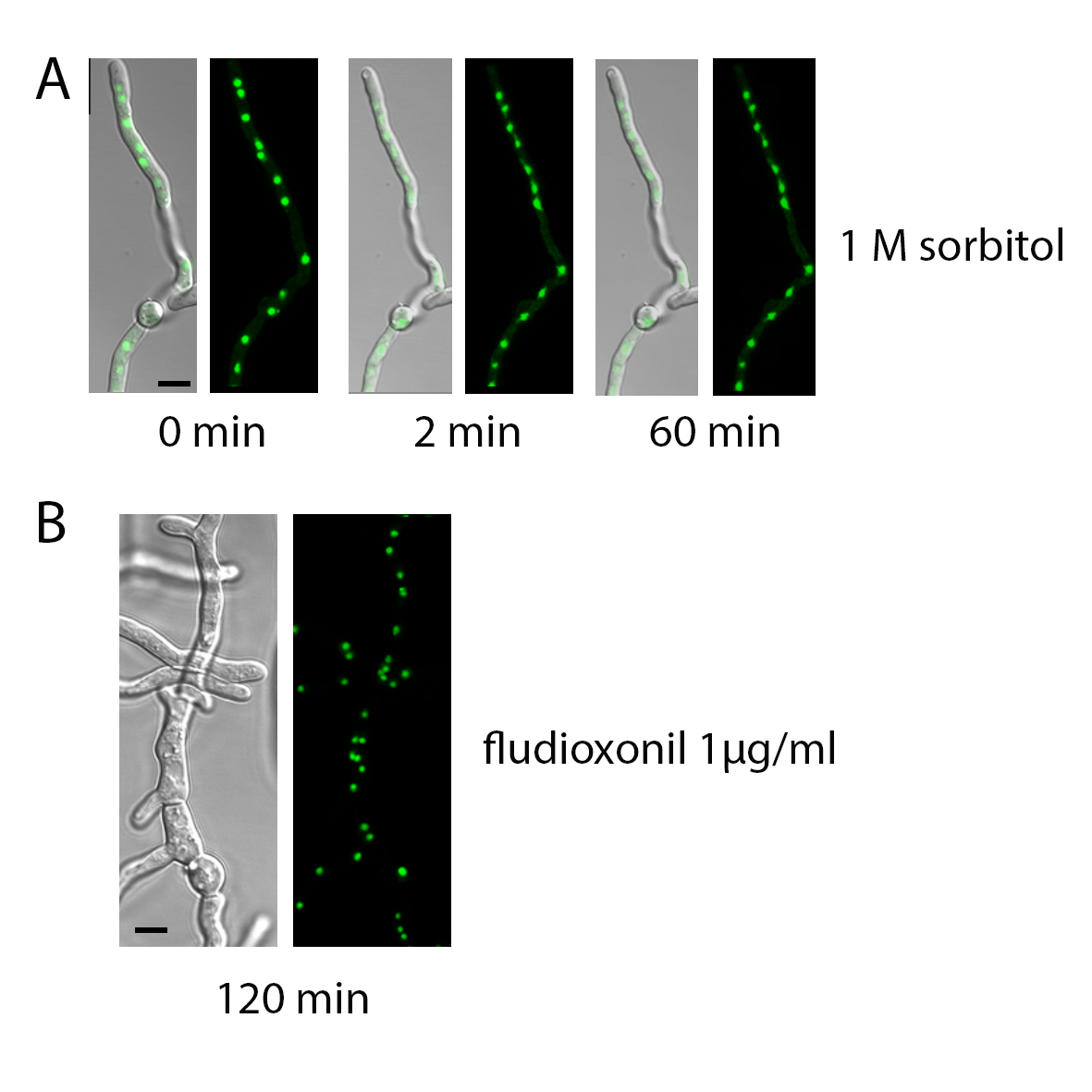

Supplement: Fig. S3 — Localization GFP-Afu5g05710. [file mbio.01184-24-s0003.tif]

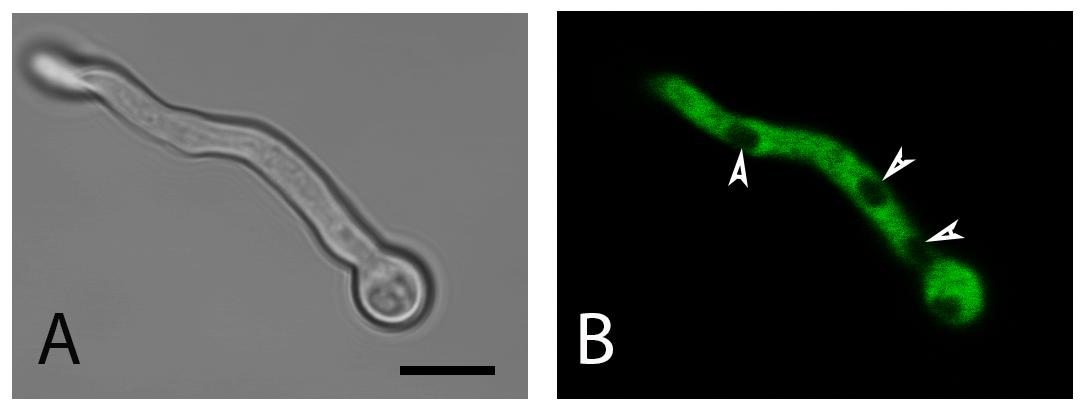

Supplement: Fig. S4 — GFP-TcsC210-1337 is not recruited to the nucleus. [file mbio.01184-24-s0004.tif]
