# Supplementary material for: The N-terminus of the Aspergillus fumigatus group III hybrid histidine kinase TcsC is essential for its physiological activity and targets the protein to the nucleus
Source: mBio. 2024 Jun 4;15(7):e01184-24. doi: 10.1128/mbio.01184-24 (PMC11253588; doi:10.1128/mbio.01184-24)
Supplement: Legends — for supplemental figures. [file mbio.01184-24-s0005.docx]

Figure S1: Targeting of TcsC proteins to the nucleus. The panels show fluorescence images and the corresponding bright field images of the ∆*tcs*C mutant expressing GFP-TcsC (panel A) and the AfS35 wild type strain expressing TcsC-GFP (panel B). The images show maximum projections of confocal stacks and corresponding bright field micrographs. Bars in the bright field images indicate 5 µm each and are also valid for the corresponding fluorescence images.

Figure S2: A truncated TcsC moiety lacking the signaling module shows a nuclear targeting, but no redistribution in response to fludioxonil. Untreated control hyphae of an AfS35 strain expressing GFP-TcsC_1-749_ are shown in panels A and B, the same hyphae after 120 min in the presence of fludioxonil (1 µg/ml) are depicted in C and D. Panels A and C show bright field images and B and D the corresponding GFP fluorescence. The GFP micrographs are maximum projections of stacks of confocal images and the bright field images show only one optical plane. The bar in A represents 5 µm and is valid for all panels.

Figure S3: Localization GFP-Afu5g05710 in *A. fumigatus* strain AfS35. Images were taken at the indicated time points after treatment with either 1 M sorbitol or 1 µg/ml fludioxonil. They represent maximum projections of stacks of confocal images and the corresponding bright field images. Bars indicate 5 µm.

Figure S4: A GFP-TcsC_210-1337_ fusion protein is not recruited to the nucleus. Hyphae were grown in the presence of 30 µg/ml doxycycline to induce expression of the fusion protein. A bright field image and the corresponding GFP micrograph showing a single confocal image are depicted in panels A and B, respectively. The bar in A represents 5 µm and is also valid for B. Several nuclei in B are indicated by arrowheads.
