# Supplementary material for: The N-terminus of the Aspergillus fumigatus group III hybrid histidine kinase TcsC is essential for its physiological activity and targets the protein to the nucleus
Source: mBio. 2024 Jun 4;15(7):e01184-24. doi: 10.1128/mbio.01184-24 (PMC11253588; doi:10.1128/mbio.01184-24)
Supplement: Table S1 — Strains used in this study. [file mbio.01184-24-s0006.docx]

| **Designation** | **Source** |
| --- | --- |
| *A. fumigatus* AfS35 | (26) |
| *A. fumigatus* AfS35 Δ*tcs*C | (7) |
| *A. fumigatus* AfS35 Δ*tcs*C + *tcs*C | (7) |
| *A. fumigatus* AfS35 *gfp*-*tcs*C_210-1337_ | (16) |
| *A. fumigatus* AfS35 *gfp-tcs*C | this study |
| *A. fumigatus* AfS35 *gfp-tcs*C + *fibrillarin-rfp* | this study |
| *A. fumigatus* AfS35 *tcsC*-*gfp* | this study |
| *A. fumigatus* AfS35 *gfp*-*tcs*C + *rfp*-*stu*A | this study |
| *A. fumigatus* AfS35 Δ*tcs*C *gfp*-*tcs*C | this study |
| *A. fumigatus* AfS35 *gfp*-Afu5g05710 | this study |
| *A. fumigatus* AfS35 Δ*tcs*C + *tcs*C_210-1337_ | this study |
| *A. fumigatus* AfS35 *tcs*C_1-208_-*gfp* | this study |
| *A. fumigatus* AfS35 *tcs*C_1-162_-*gfp* | this study |
| *A. fumigatus* AfS35 *tcs*C_62-208_-*gfp* | this study |
| *A. fumigatus* AfS35 *tcs*C_71-208_-*gfp* | this study |
| *A. fumigatus* AfS35 *tcs*C_1-208_^Δ66-69^-*gfp* | this study |
| *A. fumigatus* AfS35 *tcs*C_1-208_^Δ127-137^-*gfp* | this study |
| *A. fumigatus* AfS35 *tcs*C_1-208_^Δ145-158^-*gfp* | this study |
| *A. fumigatus* AfS35 Δ*tcs*C + tcsC_210-1335_ | this study |
| *A. fumigatus* AfS35 *gfp*-*tcs*C^R129D^ | this study |
| *A. fumigatus* AfS35 *gfp*-*tcs*C^D155H^ | this study |
| *A. fumigatus* AfS35 Δ*tcs*C + *tcs*C^R129D^ | this study |
| *A. fumigatus* AfS35 Δ*tcs*C + *tcs*C^D155H^ | this study |
| *A. fumigatus* AfS35 + sakA-*gfp* | this study |
| *A. fumigatus* AfS35 + sakA*- *gfp* | this study |
| *A. fumigatus* AfS35 Δ*tcs*C + *tcs*C_210-1335_ + *sak*A- *gfp* | this study |
| *A. fumigatus* AfS35 Δ*tcs*C + tcsC^R129D^ + *sak*A- *gfp* | this study |

**Supplementary Table 1: Strains used in this study**. The numbers in subscript and superscript refer to amino acid residues of the respective protein. sakA*: the phosphorylation site TGY at positions 170 to 173 of SakA was mutated to AGA.
