# Supplementary material for: The N-terminus of the Aspergillus fumigatus group III hybrid histidine kinase TcsC is essential for its physiological activity and targets the protein to the nucleus
Source: mBio. 2024 Jun 4;15(7):e01184-24. doi: 10.1128/mbio.01184-24 (PMC11253588; doi:10.1128/mbio.01184-24)
Supplement: Table S2 — Oligonucleotides used in this study. [file mbio.01184-24-s0007.docx]

| **Designation** | **Sequence** |
| --- | --- |
| StuA in EcoRV-FOR | ATGTCCTACACCTACACCA |
| StuA in EcoRV-REV | TCATCGACGACGAGGCATG |
| fibrillarin FORinNdeI | CATGGCATTCGGTGGTCCTAGA |
| fibrillarin_REVohneStop | GCTGCAGAACGCTTGTAGATACC |
| TcsC PmeI-REV | CCTTCTACGGCCTTTGGAGAGCG |
| TcsC-START-FOR | ATGACTGGCGCAGACGAGACGCT |
| TcsC-STOP-REV | GCTACGGCCTTTGGAGAGCGAT |
| Afu5g05710-FOR | GATGGCAGTCTCAGTGACTAAG |
| Afu5g05710-REV | CTAGTCGCTGTCATCACTGTC |
| GFP-HAMP1-FOR | GATGGTTGAGAATGAAGATGTAGTC |
| TcsC AA 1-208 (PmeI)-REV | CCCAATGCCCGTCGCGTGTG |
| TcsC 1-162 in EcoRV-FOR | GATGACTGGCGCAGACGAGA |
| TcsC 1-162 in EcoRV-REV | CTATCGAGTTCTCGAGTCTACATCATCATC |
| TcsC ab 63 mit ATG | ATGGCTTTAATTCGACGTGTTCGC |
| TcsC ab 71 mit ATG | ATGCTAGAGTTTCAAGTTGTCAGTCATC |
| Deletion AA 127-137 TcsC-FOR | GAAGCGGAAGACAATGAG |
| Deletion AA 127-137 TcsC-REV | TGAAGTTTCCTGCTGCATG |
| Deletion AA 145-158 TcsC-FOR | TCGAGAACTCGACTCGTTC |
| Deletion AA 145-158 TcsC-REV | AGCCTCATTGTCTTCCGC |
| Sub AA 129 TcsC R-D FOR | TTCAAGACGTGATCAGAGAACAAGGAGAATCCG |
| Sub AA 129 TcsC R-D REV | GTTTCCTGCTGCATGCCG |
| TcsC Sub. AS 155 (D-H)-REV | TCGTCATCGAGCGCCTCA |
| TcsC Sub. AA 155 (D-H)-FOR | CGACAGTGATCATGATGTAGACTCGAGAA |
| TcsC RRVR Rev | AATTAAAGCCTCCAACTCAAATTCAAATGC |
| TcsC RRVR For | CATCTAGAGTTTCAAGTTGTCAGTCATC |
| TcsC-upstream-FOR | TCCAACTCACAACTCAATCG |
| TcsC-SfiI-up-REV | TGTGTCTGTTGTAAATTAGTTAAC |
| TcsC-SfiI-down-REV | ATGCCGGCCATCTAGGCCGAAGACAATCGCTTATTGGAC |
| TcsC-downstream-REV | CGCGGTAGGGTACAAAAAGAA |
| SakA-FOR | ATGGCCGAGTTCGTGCGT |
| SakA-RFP-REV | GCTGCATAGTTTTGTTGCGC |
| SakA-TGY-AGAexchFOR | AGATGgccGGCgctGTTTCAACGA |
| SakA-TGY-AGAexchREV | GAGGGTCTTGGATACGAGCAAGG |
| hph-rev | TTCCTTTGCCCTCGGACGAG |
| pSilent hyg-FOR | GCCGCGACGTTAACTGATATT |
| hygromycin-REV | ATCGGGAGCGCGGCCGAT |

**Suppl. Table 2: Oligonucleotides used in this study.** Restriction sites are underlined.
